# Thermal Decomposition Behavior and Thermal Safety of Nitrocellulose with Different Shape CuO and Al/CuO Nanothermites

**DOI:** 10.3390/nano10040725

**Published:** 2020-04-11

**Authors:** Ergang Yao, Ningning Zhao, Zhao Qin, Haixia Ma, Haijian Li, Siyu Xu, Ting An, Jianhua Yi, Fengqi Zhao

**Affiliations:** 1 Science and Technology on Combustion and Explosion Laboratory, Xi′an Modern Chemistry Research Institute, Xi′an 710065, China; yaoerg@126.com (E.Y.); qzhao87@163.com (Z.Q.); h.j.Li@outlook.com (H.L.); xusy99@163.com (S.X.); anting715@163.com (T.A.); npecc_yjh2819@163.com (J.Y.); 2 School of Science, Xi’an University of Technology, Xi’an 710054, China; 3 School of Chemical Engineering, Northwest University, Xi’an 710069, China; mahx@nwu.edu.cn

**Keywords:** nanoenergetic material, compatibility, nonisothermal reaction kinetics, thermal safety, catalytic action

## Abstract

Bamboo leaf-like CuO(b) and flaky-shaped CuO(f) were prepared by the hydrothermal method, and then combined with Al nanoparticles to form Al/CuO(b) and Al/CuO(f) by the ultrasonic dispersion method. The phase, composition, morphology, and structure of the composites were characterized by X-ray powder diffraction (XRD), transmission electron microscopy (TEM), scanning electron microscopy (SEM), and energy scattering spectrometer (EDS). The compatibility of CuO, Al/CuO and nitrocellulose (NC) was evaluated by differential scanning calorimetry (DSC). The effects of CuO and Al/CuO on the thermal decomposition of NC were also studied. The results show that the thermal decomposition reactions of CuO-NC composite, Al/CuO-NC composite, and NC follow the same kinetic mechanism of Avrami-Erofeev equation. In the cases of CuO and Al/CuO, they could promote the O-NO_2_ bond cleavage and secondary autocatalytic reaction in condensed phase. The effects of these catalysts have some difference in modifying the thermolysis process of NC due to the microstructures of CuO and the addition of Al nanopowders. Furthermore, the presence of Al/CuO(f) can make the Al/CuO(f)-NC composite easier to ignite, whereas the composites have strong resistance to high temperature. Compatibility and thermal safety analysis showed that the Al/CuO had good compatibility with NC and it could be used safely. This contribution suggests that CuO and Al/CuO played key roles in accelerating the thermal decomposition of NC.

## 1. Introduction

Nanosize metal oxides exhibit excellent electrical, optical, magnetic, and catalytic properties, because they have high specific surface area and surface energy, and active sites. Therefore, the preparation and application of nanosize metal oxides have attracted widespread attention. Recently, nanosize CuO has been extended studies for the potential applications in ion batteries [1,2], gas sensors [3], catalysts [4,5], magnetoelectric effects [6], etc. It has become the typical representative of nanosize transition metal oxides. In the field of explosives and solid propellants [7], nanosize CuO, as an important catalyst, has been applied for promoting combustion. There are a lot of reports regarding the catalytic effects of nanometal oxides to the main components of solid propellants [8]. CuO can decrease the decomposition peak temperature and activity energy of ammonium perchlorate (AP), and increase the decomposition reaction rate and releasing heat of AP [9,10]. When adding nanosize CuO to solid propellant formulations, it can increase the burning rate and decrease the pressure index [11].

The temperature of thermite reaction between CuO and Al can reach 2840 K, and its volume energy density is approximately three times higher than TNT [12]. The theoretical combustion heat of the Al/CuO thermite can reach to 3324.45 kJ·kg^−1^, which is higher than that of Al/ZnO (3256.33 kJ·kg^−1^), Al/CdO (2045.85 kJ·kg^−1^), and Al/Bi_2_O_3_ (1511.26 kJ·kg^−1^). When the Al/CuO nanothermites are used as a combustion catalyst, it can effectively improve the combustion performance of solid propellants due to the excellent characteristics of high energy density, high burning rate, high temperature of reaction product, and no need for oxygen during the combustion of Al/CuO nanothermites [13,14,15].

As a main energetic component, nitrocellulose (NC) with a nitrogen amount higher than 12% is widely used for gun and rocket propellants [16,17,18,19]. However, the applications of NC are extremely limited, due to the low burning temperature, the high impact sensitivity, the high friability, and the low density [20,21]. Additionally, the exothermic degradation of NC exists the potential hazard during the preparation, storage, and use [22,23]. It has been extensively studied experimentally and theoretically for revealing the pyrolysis mechanism and improving the energetic characteristics [24]. As an important research means, thermal analysis technology, such as differential scanning calorimetry (DSC) and thermogravimetric (TG), play an important role in obtaining the thermal decomposition performance of energetic materials. Additionally, the kinetic analysis that can obtain the kinetic parameters (pre-exponential factor, activation energy, and reaction model) by using DSC or TG data are also very useful for understanding the thermal decomposition reaction mechanism [25,26]. Accordingly, the non-isothermal kinetics approach of energetic materials based on the thermal analysis technique is employed to obtain the kinetic parameters of the thermal decomposition.

It is extremely importance that all the materials in the system are compatible due to the particularity of the energetic material itself. This means that they do not interact chemically and physically with each other in the energetic materials system. Poor compatibility might give rise to safety hazards in handing and deteriorated performance. Therefore, if we want to apply a novel substance or material to explosives, propellants, and pyrotechnics system, the first issue that must be considered is the compatibility of the new material with each other components. Various additives have been mixed with NC for either enhancing its stability or improving its pyrolysis properties. Current research shows that the additive, such as nanometal oxide, can accelerated the rupture of the O-NO_2_ bonds [27] of NC and generated the NO_2_ gas. A large amount of NO_2_ can be adsorbed, which further enhances the secondary autocatalytic reaction of NC, due to the high specific surface area of Cr_2_O_3_ nanoparticles [28]. The DSC method is one of the most commonly used methods, not only for evaluating the chemical compatibility between components in the mixtures system at high temperatures, but also for investigating the thermal safety characteristic and the thermal decomposition behavior of the NC with catalyst [29,30,31,32,33,34,35].

In this study, the Bamboo leaf-like CuO(b) and flaky-shaped CuO(f) were prepared by the hydrothermal method. The differential scanning calorimetry (DSC) was used to evaluate the compatibility between CuO, Al/CuO, and NC. The thermal behavior and nonisothermal decomposition kinetics of the different CuO and Al/CuO to NC was investigated and the thermal decomposition kinetic mechanism function was explored. The DSC method evaluated the thermal safety characteristic of the NC composite system with CuO or Al/CuO as catalyst, which has the advantages of cheap, small quantity of sample required, and the capability of quickly select samples with better thermal decomposition performance.

## 2. Experimental

### 2.1. Materials

CuCl_2_·2H_2_O, NaOH and anhydrous ethanol were purchased from Xilong Chemical Reagent Co. (Guangzhou, China), and they were all of analytical grade. Al nanopowders (chemical grade), approximately 50 nm in average diameter, was purchased from Jiaozuo Banlv Nano Material Engineering Co. Ltd. (Jiaozuo, China). NC (12.6% N) was obtained from Xi’an Modern Chemistry Research Institute (Xi’an, China). All of the chemicals were used without further purification. The deionized water was used in entire experiment course.

### 2.2. Preparation of Bamboo Leaf-like CuO(b) and Al/CuO(b)

0.47 g of CuCl_2_·2H_2_O was dissolved in 20 mL deionized water, and then 10 mL NaOH solution (1 mol·L^−1^) were added dropwise. After stirring for 30 min, the mixture was poured into a hydrothermal reaction vessel and reacted at 120 °C for 8 h. After the reaction is completed and natural cooling afterwards, the precipitate was separated and washed with anhydrous ethanol and deionized water several times. Subsequently, the precipitate was dried in an oven at 60 °C. Finally, the bamboo leaf-like CuO(b) was obtained.

After the CuO(b) were mixed up with Al nanopowders according to the mole rate of 1.33:1 for Al:CuO [36,37], the Al/CuO(b) nanothermite was prepared by the ultrasonic dispersion method.

### 2.3. Preparation of Flaky-shaped CuO(f) and Al/CuO(f)

0.47 g of CuCl_2_·2H_2_O was dissolved in 20 mL deionized water, and then 10 mL NaOH solution (1 mol·L^−1^) was added dropwise. After stirring for 30 min, the mixture was poured into a hydrothermal reaction vessel and reacted at 180 °C for 16 h. After the reaction is completed and natural cooling afterwards, the precipitate was separated and then washed with anhydrous ethanol and deionized water for several times. Subsequently, the precipitate was dried in an oven at 60 °C. Finally, the flaky-shaped CuO(f) was obtained.

The Al/CuO(f) nanothermite was prepared by the ultrasonic dispersion method after the CuO(f) were mixed up with Al nanopowders according to the mole rate of 1.33:1 for Al:CuO [36,37].

### 2.4. Samples Characterization

An FEI Quanta 400 (FEI Co., Hillsboro, OR, USA) field-emission environment scanning electron microscope (SEM) characterized the morphology and structure of the sample. The acceleration voltage was 30 kV and current was 4 A. OXFORD INCAIE350 energy-dispersive X-ray spectroscopy (EDS) from OXFORD Instruments INC (Oxford, UK) was used to roughly examine the composition of the sample. The discharge voltage was 4–10 kV and the distance between the electrodes was exactly 1 mm. The morphology and size of the sample were investigated via transmission electron microscopy (TEM) and high-resolution TEM using HITACHI H-7650B (Tokyo, Japan). The operating voltage was 80 kV and resolution was 0.2 nm (lattice image). The purity and phase structure of the sample were confirmed by powder X-ray diffraction (XRD) analysis on a Rigaku D/MAX-3C (Tokyo, Japan) X-ray powder Diffractometer. The radiation source was Cu *K_α_* (*λ* = 1.5418 Å) at 40 kV and 40 mA, and the range of 2*θ* was 10 ° to 80 °.

### 2.5. Measurement of Thermal Decomposition Properties and Compatibility

The thermal behavior and kinetic analysis of the samples were performed while using differential scanning calorimetry (DSC). The effects of CuO and Al/CuO on the thermal decomposition properties of nitrocellulose (NC) were investigated using DSC (Q2000, TA Co., New Castle, DE, USA) under an N_2_ atmosphere at a flow rate of 50 mL·min^−1^. The test temperature range was room temperature to 300 °C, and the heating rate was 5.0, 10.0, 15.0, 20.0, 25.0, and 30.0 °C·min^−1^. The mass of each sample was about 0.22 mg and the sample cell is an aluminum crucible. The CuO-NC and Al/CuO-NC composites were obtained by physical mixing in an agate mortar at room temperature. The mass ratio of CuO: NC for the CuO-NC composites, and Al/CuO: NC for the Al/CuO-NC composites was 1:19.

The DSC thermal analysis method was used to assess the compatibility of different CuO and Al/CuO nanothermites with NC. The results come from the above-mentioned DSC test at the heating rate of 10.0 °C·min^−1^. The evaluation standard for compatibility of ingredients can refer to [29,30].

## 3. Results and Discussion

### 3.1. Morphology and Structure

**Figure 1:**
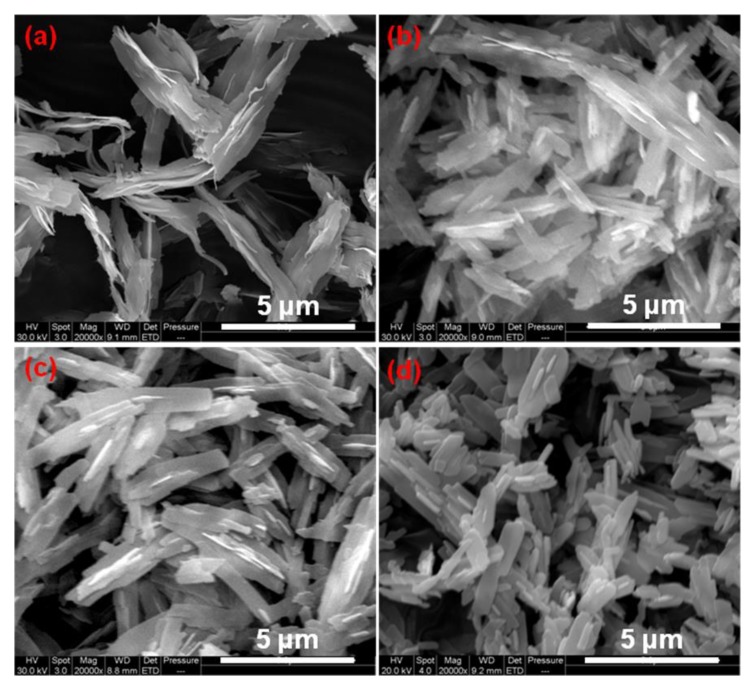
Scanning electron microscope (SEM) images of the as-prepared CuO with different reaction temperatures. (**a**) 80 °C; (**b**) 120 °C; (**c**) 160 °C; and, (**d**) 180 °C.

Figure 1 shows the SEM images of CuO prepared at different reaction temperatures. When the reaction temperature is 80 °C, the obtained CuO is spindle shaped in the whole, as shown in Figure 1a. That is thick in the middle and thin in both ends. There are many stripe protrusions in the middle spindle shaped structure, and the ends of spindle shaped structure are curly. When the reaction temperature is 120 °C, the obtained CuO is bamboo leaf-shaped (Figure 1b). There are a few protrusions on the surface, and the leaves are thin. When the reaction temperature is 160 °C, the obtained CuO is in the boat form (Figure 1c). Its size becomes smaller than the structure that is shown in Figure 1a,b. When the reaction temperature is 160 °C, the size of obtained CuO is the smallest, and there are raised fragments on the surface (Figure 1d). These experimental results show that the size of the obtained CuO gradually decreases with the temperature increases and its morphology also obviously changed.

**Figure 2:**
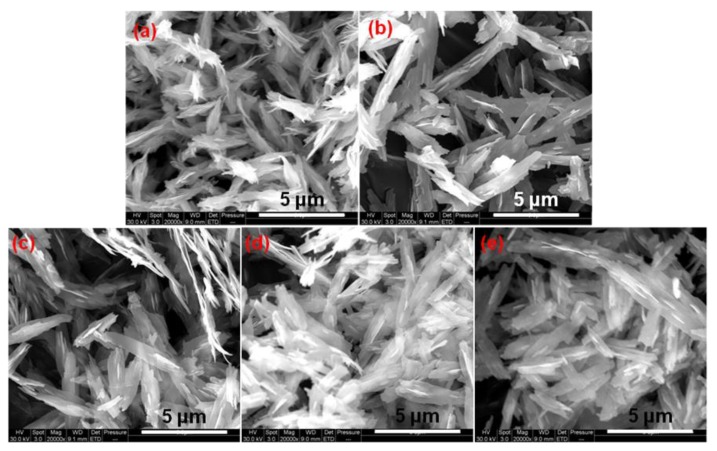
SEM images of the as-prepared CuO at 120 °C with different reaction times. (**a**) 2 h; (**b**) 4 h; (**c**) 8 h; (**d**) 12 h; and, (**e**) 16 h.

Figure 2 shows the SEM images of CuO prepared at 120 °C with different reaction times. When the reaction time is 2 h, the structure of obtained CuO is similar to the “shrimp head” with multiple curled “shrimp beard” at the end, as shown in Figure 2a. When the reaction time is 4 h, most of the obtained CuO structures are bamboo leaf-shaped (Figure 2b). There are a lot of protrusions on the surface, and the leaves are thin. When the reaction time is 8 h, the obtained CuO is also bamboo leaf-shaped (Figure 2c). There are a few protrusions at the middle region of surface, and the two ends are flaky-shaped. As the reaction time extend to 12 h (Figure 2d) or 16 h (Figure 2e), the morphology of the obtained CuO is found not significantly different from the CuO that is shown in Figure 2c. However, the agglomeration degree of CuO is obviously increased. This shows that the morphology of the obtained CuO is changed obviously with the extension of the reaction time. As the reaction time exceeds 8 h, the morphology is changed very little and it has significant aggregate.

**Figure 3:**
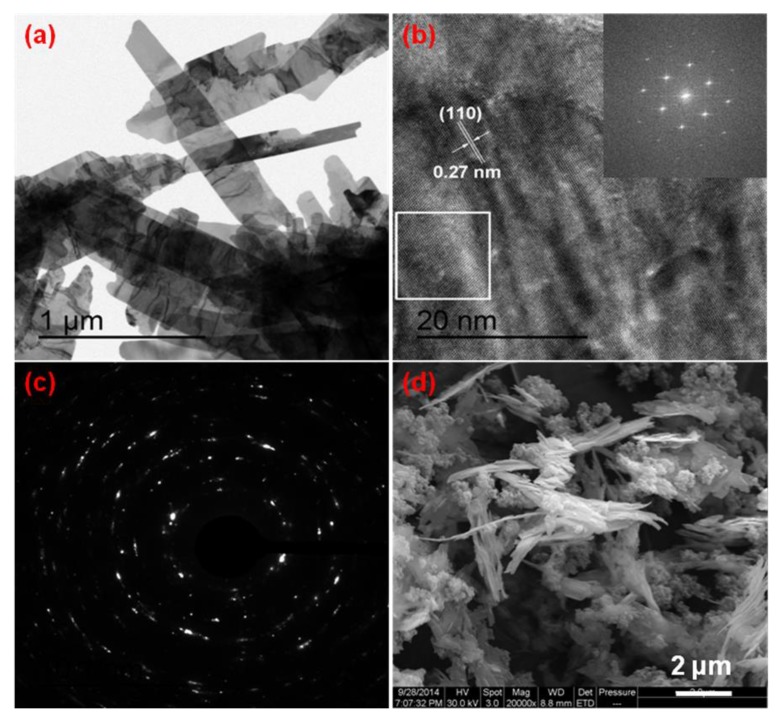
(**a**) Transmission electron microscopy (TEM) image of bamboo leaf-like CuO(b); (**b**) High-resolution transmission electron microscope (HRTEM) image of bamboo leaf-like CuO(b) and the corresponding fast Fourier transform(FFT) pattern (insert); (**c**) Selected area electron diffraction (SAED) pattern of bamboo leaf-like CuO(b); and, (**d**) SEM image of Al/CuO(b) nanothermites.

TEM and SEM analysis were performed to probe the elemental composition and morphology of the bamboo leaf-like CuO(b) obtained by reacting at 120 °C for 8 h and corresponding Al/CuO nanothermites, the results are exhibited in Figure 3. It can be seen from the TEM image of bamboo leaf-like CuO(b) (Figure 3a) that the maximum leaf of CuO(b) is about 1.6 μm in length, 300 nm in width, and 57 nm in thickness. The leaf surface is not smooth. There are many folds and a few leaves have protrusions in the middle. The protrusion is about 25 nm wide and about 90 nm long. Figure 3b is a high-resolution transmission electron microscope (HRTEM) image of the CuO and its corresponding fast Fourier transform (FFT) pattern. It is found that the lattice fringes with a lattice distance of 0.27 nm appeared at this position, corresponding to the (110) plane. Figure 3c is the selected area electron diffraction (SAED) image of the CuO sample. From Figure 3c, one can see that there are some clear scattered points, which indicate that the CuO leaves are single crystal structure and well-crystallized. Figure 3d is the SEM image of Al/CuO nanothermites. The spherical Al nanopowders and bamboo leaf-like CuO stick to each other, as shown in Figure 3d. Additionally, there are some agglomerate for the Al nanopowders because of their small size.

**Figure 4:**
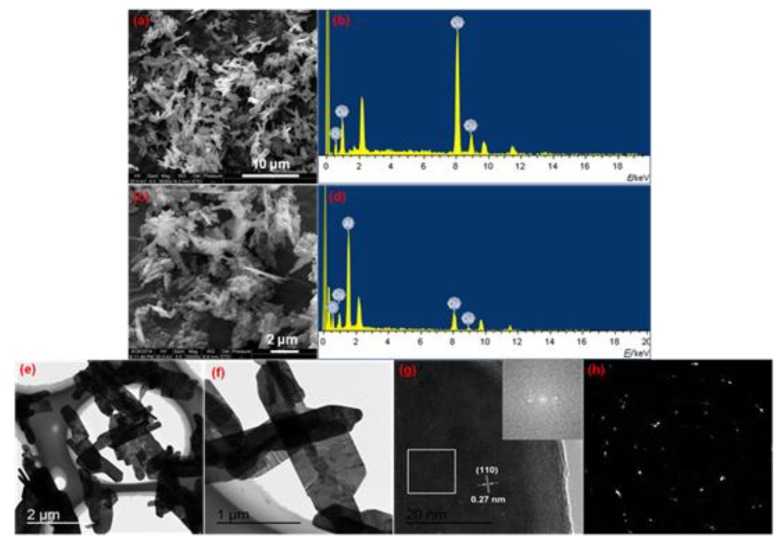
(**a**) SEM image of flaky-shaped CuO(f); (**b**) Energy-dispersive X-ray spectroscopy (EDS) pattern of flaky-shaped CuO(f); (**c**) SEM image of Al/CuO(f) nanothermites; (**d**) EDS pattern of Al/CuO(f) nanothermites; (**e**,**f**) are TEM images of flaky-shaped CuO(f); (**g**) HRTEM image of flaky-shaped CuO(f) and the corresponding FFT pattern (insert); and, (**h**) SAED pattern of flaky-shaped CuO(f).

**Figure 5:**
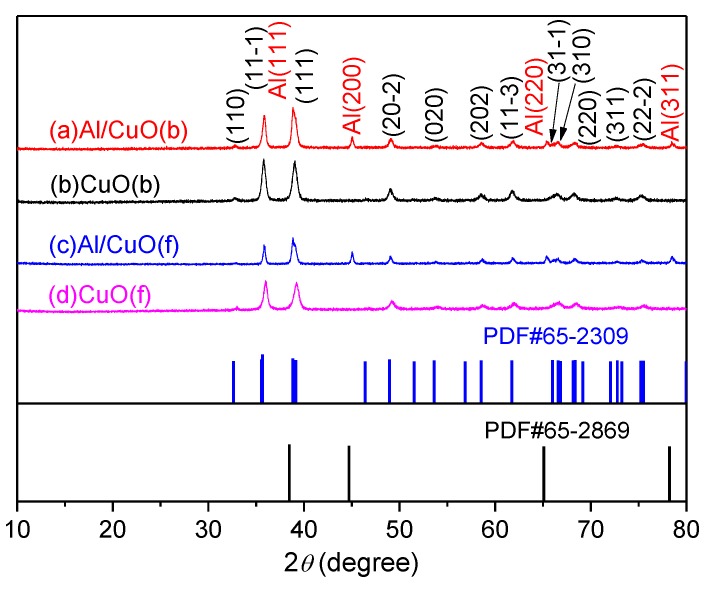
X-ray diffraction (XRD) patterns of different CuO and Al/CuO nanothermites. (**a**) Al/CuO(b); (**b**) CuO(b); (**c**) Al/CuO(f); and, (**d**) CuO(f).

We present their SEM images and energy-dispersive X-ray spectroscopy (EDS) patterns to analyze the morphology and composition of the above flaky-shaped CuO(f) and its corresponding Al/CuO(f) nanothermites samples, as shown in Figure 4a–d. It can be seen from Figure 4a that the shape of as-prepared CuO is flaky-shaped, the surface is not smooth, and it is covered with depressions and protrusions. Figure 4c is the SEM image of Al/CuO(f) nanothermites sample obtained by ultrasonic dispersion method with Al nanopowders and flaky-shaped CuO(f). Some spherical aluminum nanoparticles with smaller particle sizes are coated on the surface of some CuO fragments, as shown in Figure 4c. The self-agglomeration phenomenon of the Al nanopowders is weak due to the ultrasonic effect. The EDS and XRD results of flaky-shaped CuO(f) (Figure 4b and Figure 5d) show that the sample obtained by the hydrothermal method is the pure CuO phase. Figure 4d is the EDS pattern of the Al/CuO(f) nanothermites. In combination with its XRD characterization results (Figure 5c), it can be obtained that the Al/CuO(f) sample is a mixture of Al and CuO. Because there is no other characteristic diffraction peaks, except the Al and CuO. Figure 4e is a TEM image for the flaky-shaped CuO(f). It can be seen that the fragments of flaky-shaped CuO(f) are irregular in shape and have wrinkles on the surface. The thickness is about 90 nm and the dispersion is good. From the enlarged view of the flaky-shaped CuO(f) (Figure 4f), we can see that the folds on the fragments are clear. The largest fragment in size is approximately 0.75 μm wide and about 2.5 μm long, and the protrusions on the surface are about 0.13 μm wide. Figure 4g is a HRTEM image of flaky-shaped CuO(f) and its corresponding FFT pattern (insert). It is found that the lattice fringes with a lattice distance of 0.27 nm appeared at this position, corresponding to the (110) plane. Figure 4h is a SAED pattern of the flaky-shaped CuO(f) sample. The clear scattered points presented in Figure 4h indicate that the flaky-shaped CuO(f) is a single crystal structure with well-crystallized.

The XRD was used to analyze the phase microstructure, and the results are shown in Figure 5. From the diffraction pattern, the characteristic diffraction peaks of the bamboo leaf-like CuO(b) and flaky-shaped CuO(f) observed at the 2*θ* values of 32.95, 35.84, 39.05, 49.05, 53.79, 58.71, 61.76, 66.33, 68.19, 72.77, and 75.48 ° can be assigned as the (110), (−111), (111), (−202), (020), (202), (−311), (−113), (310), (311), and (−222) planes of CuO (JCPDS No. 65-2309). Additionally, the bamboo leaf-like CuO(b) and flaky-shaped CuO(f) are attributed to the monoclinic system, space group *C*2/c(15) with *a* = 4.662 Å, *b* = 3.416 Å, *c* = 13.7495 Å, and *α* = *β* = *γ* = 90 °. The XRD pattern also reveals that there are no unknown crystalline phase and impurities in the bamboo leaf-like CuO(b) and flaky-shaped CuO(f) samples. After the bamboo leaf-like CuO(b) and flaky-shaped CuO(f) were mixed with Al nanopowders, it also presented the characteristic diffraction peaks of Al in the Al/CuO(b) and Al/CuO(f) nanothermites. The characteristic diffraction peaks correspond to the (111), (200), (220), and (311) planes of face-centered cubic structure Al (JCPDS No. 65-2869), as shown in Figure 5a,c. Additionally, there are also no other unknown crystalline phase and impurities in Al/CuO(b) and Al/CuO(f) nanothermites. This means that there is no chemical reaction between Al nanopowders and CuO.

### 3.2. Effect of CuO and Al/CuO on Thermal Decomposition of NC

**Figure 6:**
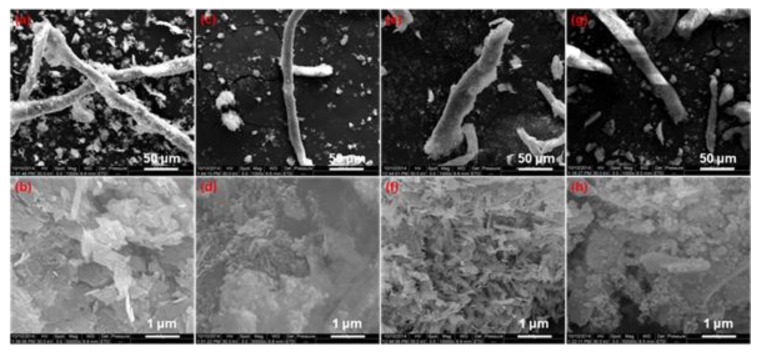
SEM images of different CuO-nitrocellulose (NC) and Al/CuO-NC composites. (**a**,**b**) CuO(b)-NC; (**c**,**d**) Al/CuO(b)-NC; (**e**,**f**) CuO(f)-NC; and, (**g**,**h**) Al/CuO(f)-NC.

The composite materials of CuO(b)-NC, CuO(f)-NC, Al/CuO(b)-NC, and Al/CuO(f)-NC were prepared by mixing NC with CuO or Al/CuO to analyze the effect of bamboo leaf-like CuO(b), flaky-shaped CuO(f) and their corresponding Al/CuO(b), Al/CuO(f) nanothermites on the thermal decomposition properties of NC. Figure 6 shows their SEM images. The CuO(b)-NC composite has a long rod shape and a rough surface. Around the rod shape structures, there are some CuO(b) particles. As can be seen from the magnified SEM image of CuO(b)-NC (Figure 6b), the surface of the NC is rough, and the bamboo leaf-shaped CuO(b) is adherent to the surface. The Al/CuO(b)-NC composite showed to be rod-shaped, and the bulk Al/CuO(b) grains are dispersed on the region near the NC short fibers, as shown in Figure 6c. It can be seen from Figure 6d that the Al/CuO(b) is adherent to the surface of the NC fibers, and there is some agglomeration on the spherical Al nanopowders and CuO fragments. The shape of CuO(f)-NC composite is rod-shaped or block-shaped with different length and size, as shown in Figure 6e. The magnified image of CuO(f)-NC (Figure 6f) shows that the surface of the NC is rough and a large number of CuO fragments adherent on the surface. The SEM images of Al/CuO(f)-NC is similar to the Al/CuO(b)-NC, and the structure is a short rod shape and the surface is rough, as shown in Figure 6g. There are also some agglomerations on the spherical Al nanopowders and CuO fragments (see Figure 6h).

#### 3.2.1. Compatibility Analysis

**Figure 7:**
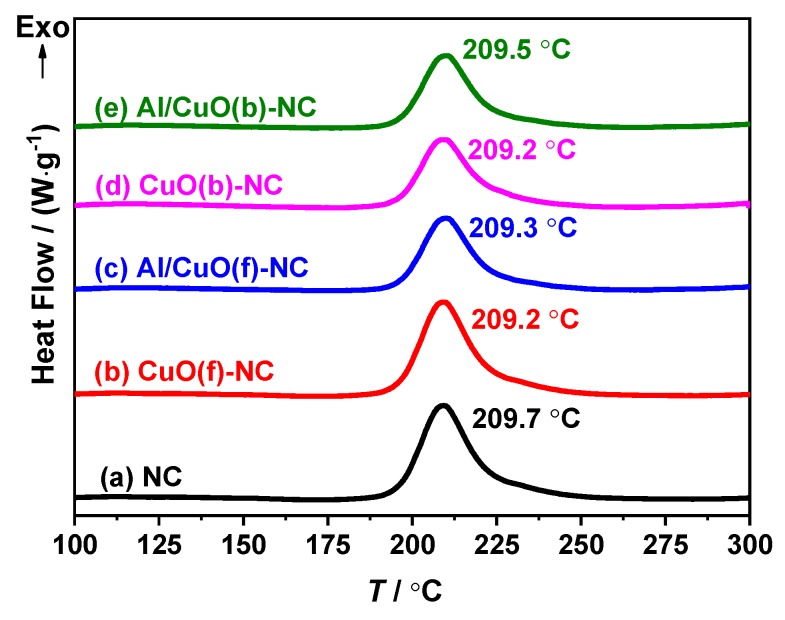
Differential scanning calorimetry (DSC) curves of NC, CuO(b)-NC, Al/CuO(b)-NC, CuO(f)-NC, and Al/CuO(f)-NC obtained at a heating rate of 10 °C·min^−1^.

Figure 7 is the DSC experimental result of NC, CuO(b)-NC, Al/CuO(b)-NC, CuO(f)-NC, and Al/CuO(f)-NC at a heating rate of 10 °C·min^−1^. There is only one exothermic peak in the thermal decomposition process of the five materials, and their peak temperatures are 209.7 °C, 209.2 °C, 209.5 °C, 209.2 °C, and 209.3 °C. When compared with the NC, the thermal decomposition peak temperatures of CuO(b)-NC, CuO(f)-NC, Al/CuO(b)-NC, and Al/CuO(f)-NC are lower than that of NC at 0.5 °C, 0.1 °C, 0.5 °C, and 0.4 °C, respectively. These results indicate that there is no reaction at a low temperature between NC and other Al and CuO reactants, and the compatibility of the bamboo leaf-like CuO(b), flaky-shaped CuO(f), Al/CuO(b), Al/CuO(f) with NC is good. Therefore, the composites can be used as a component in the preparation of propellants and explosives.

**Figure 8:**
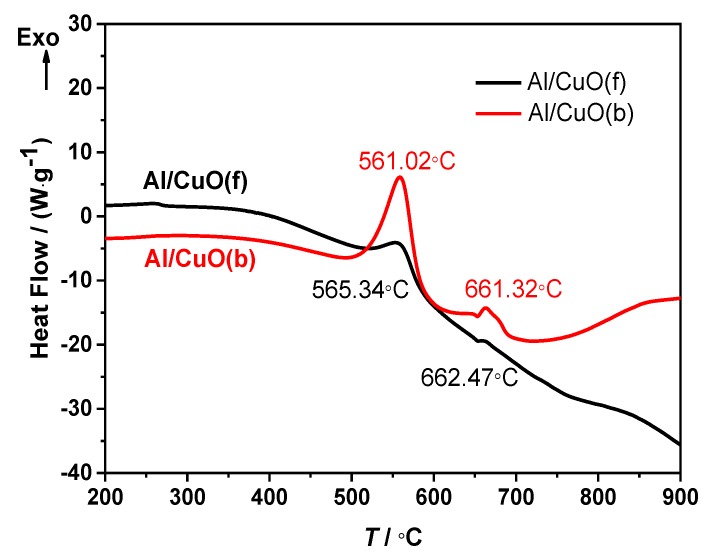
DSC curves of Al/CuO(f) and Al/CuO(b) at a heating rate of 10 °C·min^–1^.

To obtain a better understanding of the effect of the thermite reaction between Al nanopowders and CuO to the thermal decomposition of NC, the thermal reaction characteristics of Al/CuO(b) and Al/CuO(f) were investigated by DSC. Figure 8 shows the DSC curves for Al/CuO(b) and Al/CuO(f) nanothermites at a heating rate of 10 °C·min^−1^. One can see that the exothermic peak temperature of and Al/CuO(f) nanothermites are almost the same, but the releasing heat per unit mass of Al/CuO(b) (1153 J·g^1^) is obviously higher than the Al/CuO(f) (681.5 J·g^1^). The weak endothermic peaks were observed at about 654 °C from the DSC curves of Al/CuO(b) and Al/CuO(f) nanothermites. These are the melting peak of Al. The molten aluminum continues to react with CuO. The releasing heat per unit mass of the Al/CuO(b) after the melting of aluminum is approximately 101.0 J·g^1^, and the Al/CuO(f) is very little. It can also see that the temperature of main exothermic peak for Al/CuO(b) and Al/CuO(f) nanothermites is far lower than the melting temperature of aluminum. The main exothermic peak temperatures of the thermite reaction are also much higher than the thermal decomposition of CuO-NC and Al/CuO-NC, as shown in Figure 7. This can further explain there being no reaction at low temperature between NC and other Al or Al/CuO.

#### 3.2.2. Non-isothermal Kinetic Analysis

In order to explore the reaction mechanism of the intense exothermic decomposition process of NC, CuO(b)-NC, CuO(f)-NC, Al/CuO(b)-NC, and Al/CuO(f)-NC, the thermal decomposition reaction kinetics was investigated by the non-isothermal DSC method. The kinetic parameters, apparent activation energy (*E*_a_), pre-exponential factor (*A*), and kinetic model *f*(*α*) were obtained. From the non-isothermal DSC curves at different heating rates 5.0, 10.0, 15.0, 20.0, 25.0, and 30.0 °C∙min^−^^1^, one can obtained the values of the extent of conversion (*α*) to corresponding temperature (*T*) by integrating the peak area of DSC curves at different heating rates. The *E_α_* can be obtained from the slope of the liner plot of lg*β_i_* versus *T_i_* in the isoconversional Flynn-Wall-Ozawa’s method (Equation (1)) [38]. Subsequently, the values of *E_α_* to *α* can be obtained by repeating the procedure for a set of different *α*.

(1) $$\mathrm{lg}\beta_{i}=\mathrm{lg}\left[\frac{AE_{\alpha }}{RG\left(\alpha \right)}\right]-2.315-0.4567\frac{E_{\alpha }}{RT_{\alpha ,\;i}}\;i=1,\;2,\;3,\;4,\;5,\;6$$

where, *β* is heating rate (K·min^−1^); *A* is pre-exponential factor (s^−1^); *α* is the extent of conversion*; E_α_* is activation energy (J·mol^−1^) at *α*; *R* is universal gas constant (8.314 J·mol^−1^·K^−1^); *G*(*α*) is the integral form of the reaction model; and, *T* is temperature (K). Some of the reaction models used in the non-isothermal kinetic analysis are listed in the literature [39].

**Figure 9:**
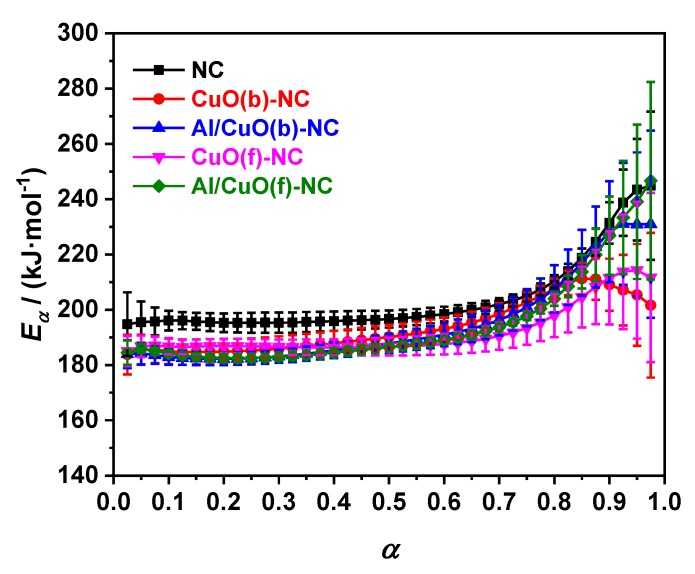
*E*_α_ vs *α* curves of NC, CuO(b)-NC, Al/CuO(b)-NC, CuO(f)-NC, and Al/CuO(f)-NC by the isoconversional Flynn-Wall-Ozawa’s method.

The isoconversional methods (i.e. Flynn-Wall-Ozawa’s method) do not need to know the reaction model, and the more accurate activation energy can also be obtained by using a set of non-isothermal curves under different heating rates [40,41]. Figure 9 shows the dependences of *E_α_* to *α* for NC, different CuO-NC, and Al/CuO-NC. The isoconversional results of *E_α_* to *α* are important in detecting and treating the multistep kinetics. The *E*_α_-*α* curves of the decomposition process for the five samples have almost the same characteristic, as can be seen from Figure 9. The activation energies of NC, different CuO-NC, and Al/CuO-NC have little changes in the range of 0.100~0.750. Additionally, the ranges can be selected to calculate the non-isothermal reaction kinetics parameters.

In order to obtain the kinetic parameters, six integral methods (MacCallum-Tanner (Equation (2)), Šatava-Šesták (Equation (3)), Agrawal (Equation (4)), General integral (Equation (5)), Universal integral (Equation (6)), Ozawa (Equation (7)) and one differential method (Kissinger (Equation (8))) were employed. By substituting the *α*-*T* data and forty-one types of kinetic model functions into the integral and differential equations, the apparent activation energy (*E*_a_), pre-exponential factor (*A*), and the most probable kinetic model *f*( *α*) were obtained by the logical choice method with the best linear correlation coefficient (*r*) [27]. Generally, the normal range of *E*_a_ and lg(*A*/s^−1^) for energetic materials is approximately 80 to 250 kJ∙mol^−1^ and 7 to 30, respectively. The values of *E*_a_, lg(*A*/s^−1^), and corresponding linear correlation coefficient (*r*) that was obtained by different methods at different heating rates are listed in Table 1 and Table 2. The values of *E*_a_ and lg(*A*/s^−1^) obtained from each single non-isothermal DSC curve is in good agreement with the calculated values that were obtained by Kissinger’s method and Ozawa’s method. Therefore, one can conclude that the reaction mechanism of the intense exothermic decomposition process of NC, CuO(b)-NC, CuO(f)-NC, Al/CuO(b)-NC, and Al/CuO(f)-NC is classified as Avrami-Erofeev equation: *f*(α) = 3/2(1−*α*)[−ln(1−*α*)]^1/3^ (differential form) and *G*(*α*) = [−ln(1−*α*)]^2/3^ (integral form).

(2) $$\mathrm{lg}G\left(\alpha \right)=\mathrm{lg}\left(\frac{AE_{a}}{\beta R}\right)-0.4828E_{a}{}^{0.4357}-\frac{0.449+0.217E_{a}}{0.001}\frac{1}{T}$$

(3) $$\mathrm{lg}G\left(\alpha \right)=\mathrm{lg}\left(\frac{AE_{a}}{\beta R}\right)-2.315-0.4567\frac{E_{a}}{RT}$$

(4) $$\ln\left[\frac{G\left(\alpha \right)}{T^{2}}\right]=\ln\left[\frac{AR}{\beta E_{a}}\frac{1-2\left(\frac{RT}{E_{a}}\right)}{1-5{\left(\frac{RT}{E_{a}}\right)}^{2}}\right]-\frac{E_{a}}{RT}$$

(5) $$\ln\left[\frac{G\left(\alpha \right)}{T^{2}}\right]=\ln\left[\frac{AR}{\beta E_{a}}\left(1-\frac{2RT}{E_{a}}\right)\right]-\frac{E_{a}}{RT}$$

(6) $$\ln\left[\frac{G\left(\alpha \right)}{T-T_{0}}\right]=\ln\left(\frac{A}{\beta }\right)-\frac{E_{a}}{RT}$$

(7) $$\mathrm{lg}\beta =\mathrm{lg}\left[\frac{AE_{\mathrm{eO}\;(\mathrm{or}\;\mathrm{pO})}}{RG\left(\alpha \right)}\right]-2.315-0.4567\frac{E_{\mathrm{eO}\;(\mathrm{or}\;\mathrm{pO})}}{RT_{e\;(\mathrm{or}\;p)}}$$

(8) $$\ln\left(\frac{\beta }{T_{p}^{2}}\right)=\ln\left(\frac{AR}{E_{K}}\right)-\frac{E_{K}}{RT_{p}}$$

where, *β* is heating rate (K·min^.1^); *A* is pre-exponential factor (s^−1^); *E*_a_ is apparent activation energy (J·mol^−1^); *R* is universal gas constant (8.314 J·mol^−1^·K^−1^); *G*(*α*) is the integral form of the reaction model; *α* is the extent of conversion*; T* is temperature (K); *T*_0_ is the initial temperature (K) at which DSC curve deviates from the baseline of the non-isothermal DSC curve; *T*_e_ is the onset temperature (K); *T*_p_ is the peak temperature (K); *E*_eO_ (J·mol^−1^); and, *E*_pO_ (J·mol^−1^) are the apparent activation energy obtained from *T*_e_ and *T*_p_ by Ozawa’s method, respectively; *E*_K_ is the apparent activation energy (J·mol^−1^) obtained from *T*_p_ by Kissinger’s method.

Substituting the *E*_a_ and *A* values (listed in Table 1 and Table 2) of NC, CuO(b)-NC, Al/CuO(b)-NC, CuO(f)-NC, Al/CuO(f)-NC, and *f*(α)=3/2(1−*α*)[−ln(1−*α*)]^1/3^ into Equation (9) [42]. The kinetic equations of the intense exothermic decomposition process of NC, CuO(b)-NC, CuO(f)-NC, Al/CuO(b)-NC, and Al/CuO(f)-NC can be described as Equations (10)–(14), respectively.

(9) $$\frac{d\alpha }{dT}=\frac{A}{\beta }f\left(\alpha \right)\exp\left(-\frac{E}{RT}\right)$$

(10) $$\frac{d\alpha }{dT}=\frac{10^{20.4}}{\beta }(1-\alpha ){\left[-\ln(1-\alpha )\right]}^{1/3}\exp(-2.5\times 10^{4}/T)$$

(11) $$\frac{d\alpha }{dT}=\frac{10^{18.0}}{\beta }(1-\alpha ){\left[-\ln(1-\alpha )\right]}^{1/3}\exp(-2.2\times 10^{4}/T)$$

(12) $$\frac{d\alpha }{dT}=\frac{10^{17.6}}{\beta }(1-\alpha ){\left[-\ln(1-\alpha )\right]}^{1/3}\exp(-2.2\times 10^{4}/T)$$

(13) $$\frac{d\alpha }{dT}=\frac{10^{18.9}}{\beta }(1-\alpha ){\left[-\ln(1-\alpha )\right]}^{1/3}\exp(-2.3\times 10^{4}/T)$$

(14) $$\frac{d\alpha }{dT}=\frac{10^{17.5}}{\beta }(1-\alpha ){\left[-\ln(1-\alpha )\right]}^{1/3}\exp(-2.2\times 10^{4}/T)$$

#### 3.2.3. Thermal Safety Analysis

The simple model derived earlier in the frame of Semenov’s thermal explosion theory [43] was established for estimating the thermal ignition temperature (*T*_be0_) and the critical temperatures of thermal explosion (*T*_bp0_) of as-prepared NC-based complexes. All of the calculations are based on the differential scanning calorimetry (DSC) experiments and the safety parameters, such as the thermal conductivity, particle size, mechanical properties, and pressure dependence of melting point et al., are not involved [44,45].

The values of *T*_e0_ and*T*_p0_ can be calculated in accordance with the Equation (15) in order to obtain the self-accelerating decomposition temperature (*T*_SADT_) of as-prepared samples. Then the value of *T*_SADT_ can be obtained by the Equation (16). The values of *T*_e0_ (*T*_SADT_) and *T*_p0_ for NC, CuO(b)-NC, CuO(f)-NC, Al/CuO(b)-NC, and Al/CuO(f)-NC are listed in Table 3.

*T*_e*i*(or p*i*)_ = *T*_e0(or p0)_ + *aβ_i_+bβ_i_*^2^*+cβ_i_*^3^ (15)

where *T*_e_ is the onset temperature; *T*_p_ is the peak temperature; *β* is the heating rate; *T*_e0_ and*T*_p0_ are the onset and peak temperatures corresponding to *β*→0, respectively; and, *a*, *b*, and *c* are the polynomial coefficients; *i*= 1, 2, ∙∙∙, 6.

*T*_SADT_=*T*_e0_ (16)

The *T*_be0_ can be obtained substituting the values of *E*_eO_ (listed in Table 1 and Table 2) and*T*_e0_ (see Table 3) into Equation (16). The *T*_bp0_ can also be obtained by substituting *E*_pO_ (listed in Table 1 and Table 2) and*T*_p0_ (see Table 3) into the Equation (17). The values of *T*_be0_ and *T*_bp0_ for NC, CuO(b)-NC, CuO(f)-NC, Al/CuO(b)-NC, and Al/CuO(f)-NC are also listed in Table 3. Generally, the value of *T*_b_ (*T*_be0_ or *T*_bp0_) is one of the most important evaluation parameters for thermal safety, which represent the degree of difficulty of the transition from thermal decomposition to thermal explosion. The higher the value of *T*_b_, the transition can take place easier.

(17) $$T_{\mathrm{be}0\;\mathrm{or}\;\mathrm{bp}0}=\frac{E_{\mathrm{eO}\;\mathrm{or}\;\mathrm{pO}}-\sqrt{E_{\mathrm{eO}\;\mathrm{or}\;\mathrm{pO}}^{2}-4E_{\mathrm{eO}\;\mathrm{or}\;\mathrm{pO}}RT_{e0\;\mathrm{or}\;p0}}}{2R}$$

While using *T* = *T*_p0_, *E*_a_ = *E*_K_, and *A* = *A*_K_, the values of activation entropy (Δ*S*^≠^), activation enthalpy (Δ*H*^≠^), and activation of activation free energy (Δ*G*^≠^) of the main exothermic decomposition reaction of the NC, CuO(b)-NC, CuO(f)-NC, Al/CuO(b)-NC, and Al/CuO(f)-NC are obtained by Equation (18) to Equation (20), as listed in Table 3.

(18) $$A\exp\left(-\frac{E_{a}}{RT}\right)=\frac{k_{B}T}{h}\exp\left(-\frac{\Delta G^{\neq }}{RT}\right)$$

Δ*H*^≠^ = *E*_a_ - *RT* (19)

Δ*G*^≠^ = Δ*H*^≠^ - *T*Δ*S*^≠^ (20)

where *k*_B_ is the Boltzmann constant, 1.38066× 10^−23^ J∙K^−1^; *h* is the Planck constant, 6.626 × 10^−34^ J∙s.

According to the previous studies [27,28,46], the thermal decomposition reaction of NC is a typical competition reaction between the O-NO_2_ bond rupture and the decomposition of polymer skeleton products decomposition. The NO_2_ gas is the initial decomposition product in the first step of high-temperature pyrolysis, similar to RDX. However, the NO_2_ that is produced by RDX pyrolysis is derived from the rupture of the N-NO_2_ bond [47]. However, the O-NO_2_ bond rupture is deemed to be the first step, resulting in the release of NO_2_. The NO_2_ stagnates in the polymer skeleton and then reacts with the RO• radical or its decomposition products to produce the NO, NO_2_, CO_2_, CO, H_2_O, N_2_O, HCHO, HCOOH, etc. Finally, the secondary autocatalytic reaction is significantly strengthened.

From the above calculation results, it could be derived that the addition of CuO and Al/CuO nanothermites can reduce the apparent activation energy (*E*_a_), pre-exponential factor (*A*), onset temperature (*T*_e0_), thermal decomposition peak temperature (*T*_p0_), critical thermal ignition temperature (*T*_be0_), and the critical temperatures of thermal explosion (*T*_bp0_), as compared with single-component NC. This indicates that the CuO and Al/CuO nanothermites can accelerate the thermal decomposition of NC. When compared with the CuO-NC composite system, the *E*_a_, *A*, *T*_e0_, *T*_p0_, and *T*_be0_ values of the Al/CuO-NC composite system are all decreased. It indicates that the addition of Al nanopowders can increase the reactive sites and then accelerate the thermal decomposition of NC because of the high specific surface area of Al nanopowders or CuO. The physical adsorption might occur between the Al nanopowders or CuO and decomposition product of nitrocellulose. This may further effect the autocatalytic decomposition reaction of nitrocellulose.

From Table 1 and Table 2, one can find that the *E*_a_ values of the thermal decomposition processes of Al/CuO(b)-NC and Al/CuO(f)-NC are 181.1 kJ∙mol^−1^ and 180.6 kJ∙mol^−1^, and the *A* values are 10^17.4^ s^−1^ and 10^17.3^ s^−1^, respectively. The little difference of *E*_a_ and *A* between the two nanothermites, indicating that the addition of Al/CuO(b) and Al/CuO(f) may reduce the thermal decomposition energy barrier of the composites and make it easy to decompose. However, the self-accelerated decomposition temperature (*T*_SADT_) of Al/CuO(f)-NC is 152.9 °C, which is 16.4 °C lower than that of Al/CuO(b)-NC. Additionally, the thermal decomposition peak temperature (*T*_p0_) of Al/CuO(f)-NC is 7.4 °C higher than the Al/CuO(b)-NC. This shows that the decomposition reaction of Al/CuO(f)-NC is stronger than that of Al/CuO(b)-NC, but the time-consumption of the decomposition process of Al/CuO(f)-NC is long. From the results of the critical thermal ignition temperature (*T*_be0_) and the critical temperatures of thermal explosion (*T*_bp0_), one can get that the *T*_be0_ of Al/CuO(f)-NC is 17.3 °C lower than the Al/CuO(b)-NC, but *T*_pe0_ of Al/CuO(f)-NC is higher than the Al/CuO(b)-NC. This indicates that the Al/CuO(f)-NC is more easily ignited, but its decomposition rate is relatively slow. Accordingly, the Al/CuO(f)-NC has a much higher thermal stability than that of the Al/CuO(b)-NC.

## 4. Conclusions

Two kinds of nanosize CuO with different morphologies and sizes were prepared via the hydrothermal method by adjusting the reaction temperature and time. The ultrasonic composite method was used to combine the aluminum nanopowders with the as-prepared bamboo leaf-shaped CuO(b) and flaky-shaped CuO(f) to obtain the corresponding Al/CuO(b) and Al/CuO(f) nanothermites. Based on the non-isothermal decomposition kinetics, the catalytic effect of the different morphologies of CuO and corresponding nanothermites on the thermal decomposition properties of NC was investigated. The additions of CuO(b), Al/CuO(b), CuO(f), and Al/CuO(f) do not change the thermal decomposition mechanism of NC. The catalytic effect of Al/CuO nanothermites to NC is better than the CuO, and the Al/CuO(b)-NC, Al/CuO(f)-NC is easier to decompose than the CuO(b)-NC and CuO(f)-NC. The results of compatibility and thermal safety analysis show that the CuO and Al/CuO catalysts have good compatibility with NC, and the catalysts can be used safely. However, the agglomeration of Al/CuO(f) is relatively serious. The self-accelerated decomposition temperature (*T*_SADT_) and the critical thermal ignition temperature (*T*_be0_) of Al/CuO(f)-NC are the lowest, while the thermal decomposition peak temperature (*T*_p0_) and the critical temperatures of thermal explosion (*T*_bp0_) are higher. It shows that the Al/CuO(f)-NC composite material is more easily ignited after being integrated with NC and Al/CuO(f), and the thermal stability during the thermal decomposition process is also better. The CuO(b), Al/CuO(b), CuO(f), and Al/CuO(f) as the catalysts has a wide application prospect in solid propellants.

## Figures and Tables

**Table 1:** Calculated values of kinetic parameters of decomposition reaction for NC, bamboo leaf-like CuO(b)-NC, and Al/CuO(b)-NC.

Method	*β*/(°C∙min^−1^)	NC			CuO(b)-NC			Al/CuO(b)-NC		
		*E*_a_/(kJ·mol^−^^1^)	lg(*A*/s^−^^1^)	*r*	*E*_a_/(kJ·mol^−1^)	lg(*A*/s^−1^)	*r*	*E*_a_/(kJ·mol^−1^)	lg(*A*/s^−1^)	*r*
MacCallum-Tanner	5.0	208.0 ± 3.5	20.6 ± 0.4	0.9983	180.3 ± 5.3	17.5 ± 0.5	0.9927	176.2 ± 5.1	17.1 ± 0.4	0.9917
	10.0	205.4 ± 4.2	20.3 ± 0.4	0.9984	183.3 ± 5.8	17.9 ± 0.2	0.9935	184.3 ± 4.7	18.0 ± 0.3	0.9920
	15.0	209.3 ± 3.4	20.7 ± 0.4	0.9988	187.3 ± 5.7	18.3 ± 0.4	0.9930	184.2 ± 5.8	18.0 ± 0.4	0.9925
	20.0	209.3 ± 2.7	20.7 ± 0.6	0.9987	182.6 ± 4.8	17.8 ± 0.6	0.9958	184.6 ± 5.7	18.0 ± 0.2	0.9927
	25.0	211.8 ± 5.1	20.9 ± 0.4	0.9990	192.6 ± 4.8	18.9 ± 0.4	0.9934	181.7 ± 4.4	17.7 ± 0.4	0.9943
	30.0	210.1 ± 4.5	20.8 ± 0.4	0.9982	187.7 ± 4.1	18.4 ± 0.3	0.9962	182.3 ± 4.3	17.8 ± 0.3	0.9959
Šatava-Šesták	5.0	204.6 ± 3.9	20.2 ± 0.4	0.9983	178.4 ± 3.2	17.3 ± 0.6	0.9927	174.6 ± 3.7	16.9 ± 0.4	0.9917
	10.0	202.1 ± 5.2	19.9 ± 0.6	0.9984	181.2 ± 4.0	17.7 ± 0.5	0.9935	182.2 ± 6.0	17.8 ± 0.3	0.9920
	15.0	205.8 ± 5.4	20.4 ± 0.3	0.9988	185.0 ± 5.1	18.1 ± 0.3	0.9930	182.1 ± 6.7	17.8 ± 0.2	0.9925
	20.0	205.8 ± 4.2	20.3 ± 0.3	0.9987	180.6 ± 4.7	17.6 ± 0.5	0.9958	182.5 ± 6.3	17.8 ± 0.4	0.9927
	25.0	208.1 ± 3.6	20.6 ± 0.5	0.9990	190.0 ± 5.6	18.7 ± 0.3	0.9934	179.8 ± 5.3	17.5 ± 0.2	0.9943
	30.0	206.6 ± 5.6	20.4 ± 0.4	0.9982	185.4 ± 5.0	18.1 ± 0.3	0.9962	180.3 ± 4.9	17.6 ± 0.3	0.9959
Agrawal	5.0	207.2 ± 5.6	20.5 ± 0.5	0.9982	179.7 ± 4.0	17.5 ± 0.3	0.9920	175.7 ± 4.5	17.1 ± 0.3	0.9909
	10.0	204.5 ± 5.3	20.2 ± 0.6	0.9983	182.6 ± 4.8	17.8 ± 0.5	0.9929	183.6 ± 4.0	17.9 ± 0.3	0.9913
	15.0	208.4 ± 4.8	20.6 ± 0.4	0.9987	186.5 ± 5.8	18.3 ± 0.5	0.9924	183.4 ± 3.4	17.9 ± 0.2	0.9919
	20.0	208.3 ± 4.0	20.6 ± 0.6	0.9986	181.9 ± 3.5	17.8 ± 0.3	0.9954	183.8 ± 4.6	18.0 ± 0.3	0.9920
	25.0	210.7 ± 5.2	20.8 ± 0.6	0.9990	191.7 ± 4.4	18.8 ± 0.5	0.9928	180.9 ± 3.6	17.6 ± 0.2	0.9938
	30.0	209.0 ± 4.7	20.7 ± 0.6	0.9981	186.8 ± 6.7	18.3 ± 0.5	0.9958	181.5 ± 4.8	17.7 ± 0.4	0.9955
General integral	5.0	205.8 ± 6.2	19.0 ± 0.4	0.9985	178.3 ± 6.7	16.0 ± 0.6	0.9919	174.3 ± 5.3	15.6 ± 0.2	0.9908
	10.0	203.3 ± 6.08	18.7 ± 0.3	0.9983	181.3 ± 6.9	16.4 ± 0.3	0.9928	182.3 ± 5.9	16.5 ± 0.3	0.9912
	15.0	207.2 ± 4.9	19.1 ± 0.5	0.9987	185.3 ± 6.8	16.8 ± 0.5	0.9923	182.2 ± 6.1	16.5 ± 0.3	0.9918
	20.0	207.2 ± 5.3	19.1 ± 0.4	0.9986	180.7 ± 3.8	16.3 ± 0.5	0.9954	182.7 ± 5.7	16.5 ± 0.4	0.9920
	25.0	209.6 ± 6.2	19.3 ± 0.6	0.9989	190.6 ± 4.4	17.4 ± 0.3	0.9928	179.8 ± 4.3	16.2 ± 0.3	0.9937
	30.0	208.0 ± 4.7	19.2 ± 0.5	0.9981	185.8 ± 3.5	16.9 ± 0.4	0.9958	180.4 ± 5.9	16.3 ± 0.4	0.9955
Universal integral	5.0	207.2 ± 4.3	20.5 ± 0.6	0.9982	179.7 ± 6.9	17.5 ± 0.4	0.9920	175.7 ± 3.9	17.1 ± 0.2	0.9909
	10.0	204.5 ± 5.3	20.2 ± 0.3	0.9983	182.6 ± 6.5	17.8 ± 0.5	0.9929	183.6 ± 4.8	17.9 ± 0.2	0.9913
	15.0	208.4 ± 4.6	20.6 ± 0.5	0.9987	186.5 ± 5.1	18.3 ± 0.3	0.9924	183.4 ± 5.7	17.9 ± 0.4	0.9919
	20.0	208.3 ± 3.9	20.6 ± 0.4	0.9986	181.9 ± 6.1	17.8 ± 0.3	0.9954	183.8 ± 3.5	18.0 ± 0.4	0.9920
	25.0	210.7 ± 5.4	20.8 ± 0.6	0.9990	191.7 ± 6.1	18.8 ± 0.4	0.9928	180.9 ± 5.5	17.7 ± 0.2	0.9938
	30.0	209.0 ± 6.1	20.7 ± 0.4	0.9981	186.8 ± 5.6	18.3 ± 0.5	0.9958	181.5 ± 4.4	17.7 ± 0.4	0.9955
Mean		207.5 ± 4.8	20.2 ± 0.5		184.5 ± 5.2	17.8 ± 0.4		181.1 ± 5.0	17.4 ± 0.3	
Flynn-Wall-Ozawa		185.7 ± 1.9 (*E*_e__O_)		0.9998	178.7 ± 3.6 (*E*_e__O_)		0.9919	176.3 ± 5.3 (*E*_e__O_)		0.9982
		197.6 ± 6.4 (*E*_p__O_)		0.9979	187.0 ± 4.8 (*E*_p__O_)		0.9987	183.6 ± 2.6 (*E*_p__O_)		0.9996
Kissinger		199.7 ± 6.8 (*E*_K_)	19.8 ± 0.7	0.9977	188.6 ± 5.0 (*E*_K_)	18.6 ± 0.6	0.9986	185.0 ± 2.7 (*E*_K_)	18.2 ± 0.3	0.9996
Mean(*E*_eO_, *E*_pO_, *E*_K_)		194.3 ± 5.0			184.8 ± 4.5			181.6 ± 3.5		

**Table 2:** Calculated values of kinetic parameters of decomposition reaction for flaky-shaped CuO(f)-NC and Al/CuO(f)-NC.

Method	*β*/(°C∙min^−1^)	CuO(f)-NC			Al/CuO(f)-NC		
		*E*_a_/(kJ·mol^−1^)	lg(*A*/s^−^^1^)	*r*	*E*_a_/(kJ·mol^−1^)	lg(*A*/s^−1^)	*r*
MacCallum-Tanner	5.0	190.4 ± 6.6	18.6 ± 0.4	0.9917	177.6 ± 5.7	17.2 ± 0.6	0.9889
	10.0	197.2 ± 4.0	19.3 ± 0.4	0.9921	177.7 ± 3.5	17.2 ± 0.6	0.9920
	15.0	192.0 ± 5.8	18.8 ± 0.5	0.9928	176.3 ± 4.7	17.1 ± 0.5	0.9936
	20.0	202.3 ± 6.8	19.9 ± 0.6	0.9932	187.8 ± 4.9	18.4 ± 0.4	0.9952
	25.0	189.1 ± 6.1	18.4 ± 0.5	0.9925	179.9 ± 3.9	17.5 ± 0.4	0.9930
	30.0	198.0 ± 3.7	19.4 ± 0.6	0.9941	190.6 ± 5.5	18.7 ± 0.4	0.9942
Šatava-Šesták	5.0	187.9 ± 6.7	18.4 ± 0.5	0.9917	175.9 ± 3.3	17.1 ± 0.5	0.9889
	10.0	194.4 ± 5.7	19.1 ± 0.4	0.9921	176.0 ± 4.5	17.1 ± 0.2	0.9920
	15.0	189.5 ± 5.3	18.5 ± 0.5	0.9928	174.7 ± 3.3	17.0 ± 0.4	0.9936
	20.0	199.2 ± 4.1	19.6 ± 0.5	0.9932	185.5 ± 6.1	18.2 ± 0.5	0.9952
	25.0	186.7 ± 6.7	18.2 ± 0.5	0.9925	178.0 ± 6.1	17.3 ± 0.5	0.9930
	30.0	195.1 ± 5.1	19.1 ± 0.5	0.9941	188.1 ± 3.8	18.4 ± 0.3	0.9942
Agrawal	5.0	189.7 ± 4.7	18.6 ± 0.5	0.9910	177.1 ± 6.6	17.2 ± 0.3	0.9879
	10.0	196.4 ± 3.7	19.3 ± 0.6	0.9915	177.1 ± 5.4	17.2 ± 0.5	0.9913
	15.0	191.2 ± 6.4	18.7 ± 0.5	0.9921	175.6 ± 5.3	17.1 ± 0.3	0.9929
	20.0	201.3 ± 5.5	19.8 ± 0.5	0.9926	187.0 ± 3.8	18.3 ± 0.3	0.9947
	25.0	188.2 ± 5.1	18.4 ± 0.5	0.9918	179.1 ± 3.7	17.4 ± 0.5	0.9923
	30.0	196.9 ± 3.2	19.3 ± 0.4	0.9936	189.7 ± 3.5	18.6 ± 0.3	0.9937
General integral	5.0	188.4 ± 5.3	17.1 ± 0.5	0.9909	175.6 ± 4.6	15.7 ± 0.3	0.9878
	10.0	195.1 ± 4.7	17.8 ± 0.6	0.9914	175.8 ± 4.2	15.8 ± 0.3	0.9912
	15.0	190.1 ± 6.1	17.3 ± 0.5	0.9921	174.4 ± 5.3	15.6 ± 0.4	0.9929
	20.0	200.3 ± 6.5	18.4 ± 0.5	0.9925	185.9 ± 5.8	16.9 ± 0.5	0.9947
	25.0	187.2 ± 3.8	16.9 ± 0.4	0.9918	178.0 ± 5.0	16.0 ± 0.6	0.9922
	30.0	196.0 ± 5.5	17.9 ± 0.6	0.9936	188.6 ± 3.2	17.2 ± 0.4	0.9936
Universal integral	5.0	201.3 ± 4.1	19.8 ± 0.6	0.9926	177.1 ± 5.5	17.2 ± 0.4	0.9879
	10.0	196.4 ± 3.4	19.3 ± 0.4	0.9915	177.1 ± 5.0	17.2 ± 0.4	0.9913
	15.0	190.1 ± 4.0	17.3 ± 0.4	0.9921	175.6 ± 6.5	17.1 ± 0.4	0.9929
	20.0	191.2 ± 6.3	18.7 ± 0.5	0.9921	187.0 ± 6.8	18.3 ± 0.3	0.9947
	25.0	188.2 ± 3.5	18.4 ± 0.6	0.9918	179.1 ± 6.7	17.4 ± 0.3	0.9923
	30.0	196.9 ± 4.1	19.3 ± 0.5	0.9936	189.7 ± 3.4	18.6 ± 0.3	0.9937
Mean		193.5 ± 5.1	18.7 ± 0.5		180.6 ± 4.9	17.3 ± 0.4	
Flynn-Wall-Ozawa		177.8 ± 5.1 (*E*_e__O_)		0.9983	171.5 ± 6.8 (*E*_e__O_)		0.9968
		183.7 ± 8.3 (*E*_p__O_)		0.9959	185.9 ± 3.9 (*E*_p__O_)		0.9991
Kissinger		185.1 ± 8.7 (*E*_K_)	18.2 ± 1.0	0.9956	187.4 ± 4.1 (*E*_K_)	18.5 ± 0.4	0.9990
Mean(*E*_eO_, *E*_pO_, *E*_K_)		182.2 ± 7.4			181.6 ± 4.9		

**Table 3:** Calculated values of kinetic parameters of decomposition reaction for NC, CuO(b)-NC, Al/CuO(b)-NC, CuO(f)-NC, and Al/CuO(f)-NC.

Sample	*E*_a_/(kJ·mol^−1^)	lg(*A*/s^−1^)	*T*_e0_/°C	*T*_p0_/°C	*T*_be0_/°C	*T*_bp0_/°C	Δ*S*^≠^/(J∙mol^−1^∙K^−1^)	Δ*H*^≠^/(kJ∙mol^−1^)	Δ*G*^≠^/(kJ∙mol^−1^)
NC	207.5	20.2	181.8	197.0	191.4	206.7	138.4	199.7	134.6
CuO(b)-NC	184.5	17.8	176.4	189.3	180.2	193.5	91.7	188.6	146.2
Al/CuO(b)-NC	181.1	17.4	169.3	184.4	178.6	194.3	84.5	185.0	146.3
CuO(f)-NC	193.5	18.7	178.6	190.0	189.3	206.3	109.1	185.0	133.9
Al/CuO(f)-NC	180.6	17.3	152.9	191.8	161.3	201.9	83.2	187.4	148.7
